# The influence of a cognitive behavioural approach on changing patient expectations for conservative care in shoulder pain treatment: a protocol for a pragmatic randomized controlled trial

**DOI:** 10.1186/s12891-021-04588-9

**Published:** 2021-08-24

**Authors:** Heather Myers, Francis Keefe, Steven Z. George, June Kennedy, Ashley Davis Lake, Corina Martinez, Chad Cook

**Affiliations:** 1 Physical Therapist, Urbaniak Sports Sciences Institute, Duke University Health System, 3475 Erwin Rd, Durham, NC 27705 USA; 2 Professor in Psychiatry and Behavioral Sciences, Psychology and Neuroscience, Medicine, and Anesthesiology, 2200 W Main St, Suite 340, Durham, NC 27705 USA; 3 Laszlo Ormandy Distinguished Professor, Department of Orthopaedic Surgery, Duke Clinical Research Institute, 200 Morris Street, Durham, NC 27701 USA; 4 Professor, Director of Clinical Research Facilitation, Duke Doctor of Physical Therapy Division, Duke Clinical Research Institute, 311 Trent Drive, Durham, NC 27705 USA

**Keywords:** Rotator Cuff, Shoulder, Cognitive Behavioral Therapy, Expectations

## Abstract

**Background:**

Despite similar outcomes for surgery and conservative care, the number of surgeries to treat rotator cuff related shoulder pain has increased. Interventions designed to enhance treatment expectations for conservative care have been shown to improve patient expectations, but no studies have yet explored whether such interventions influence patient decisions to pursue surgery. The purpose of this randomized clinical trial is to examine the effect of an intervention designed to improve expectations of conservative care on the decision to have surgery.

**Methods:**

We will test the effectiveness of the Patient Engagement, Education, and Restructuring of Cognitions (PEERC) intervention which is intended to change expectations regarding conservative care. The PEERC intervention will be evaluated in a randomized, pragmatic “add-on” trial, to better understand the effect the intervention has on outcomes. Ninety-four (94) participants with rotator cuff related shoulder pain referred for physical therapy will be randomized to receive either impairment-based care or impairment-based care plus PEERC. Both groups will receive impairment-based conservative treatment created by compiling the evidence associated with established, effective interventions. Participants assigned to the impairment-based care *plus* PEERC condition will also receive the PEERC intervention. This intervention, informed by principles of cognitive behavioral therapy, involves three components: (1) strategies to enhance engagement, (2) education and (3) cognitive restructuring and behavioral activation. Outcomes will be assessed at multiple points between enrolment and six months after discharge. The primary outcome is patient reported decision to have surgery and the secondary outcomes are pain, function, expectations and satisfaction with conservative care.

**Discussion:**

Rotator cuff related shoulder pain is highly prevalent, and because conservative and surgical treatments have similar outcomes, an intervention that changes expectations about conservative care could alter patient reports of their decision to have surgery and ultimately could lead to lower healthcare costs and decreased risk of surgical complications.

**Trial registration:**

This study is registered as NCT03353272 at ClincialTrials.gov.

## Background

In comparative trials involving rotator cuff related shoulder pain (RCRSP), conservative interventions have yielded comparable outcomes with surgery [1–4]. However, despite the greater risks of harms, higher costs, and a high percentage of re-tears associated with a surgical approach, the number of shoulder surgeries for all forms of RCRSP pain continues to escalate [5–7]. In patients with RCRSP, pre-treatment expectations of the success of surgical and/or conservative approaches have demonstrated strong relationships with post-treatment outcomes [8–10]. The shoulder is not unique in these associations as patient expectations are known to influence treatment outcomes for cervical, low back and lower extremity disorders as well [11–14].

Patient expectations are beliefs or attitudes that include pre-treatment thoughts and beliefs regarding the need for specific treatment methods and the timing and intensity of these methods. Brief interventions designed to alter and enhance treatment expectations for conservative treatment appear to result in slight improvements in expectations [15, 16], but not outcomes. The few interventions that have been tested have methodological problems including the failure to attend to issues of treatment fidelity and reliance on overly simplistic methods for altering expectations such as a patient handout or a one-time educational program [15, 16].

To date, no studies have explored whether a cognitive-behavioral intervention can influence patient reports of their decision to pursue surgery. We posit that previous approaches to change patient expectations have had only modest effects because they do not include theory-based treatment techniques known to influence patient beliefs. Our study purpose is to test an innovative intervention to alter expectations about conservative care that is informed by principles of cognitive-behavioral theory: Patient Engagement, Education, and Restructuring of Cognitions (PEERC). The cognitive-behavioral therapy (CBT) treatment techniques that form the core of our PEERC intervention are patient-centered and are designed not only to alter expectations but also decisions to pursue surgical treatment.

### Primary and Secondary Objectives

The purpose of this randomized clinical trial is to examine the effect of PEERC, an intervention designed to improve expectations of conservative care, on the patient reports of their decision to have versus not have surgery (primary outcome). Our secondary aim is to evaluate the impact of PEERC on pain, function, expectations and satisfaction with conservative care (secondary).

### Trial Design

**Fig. 1:**
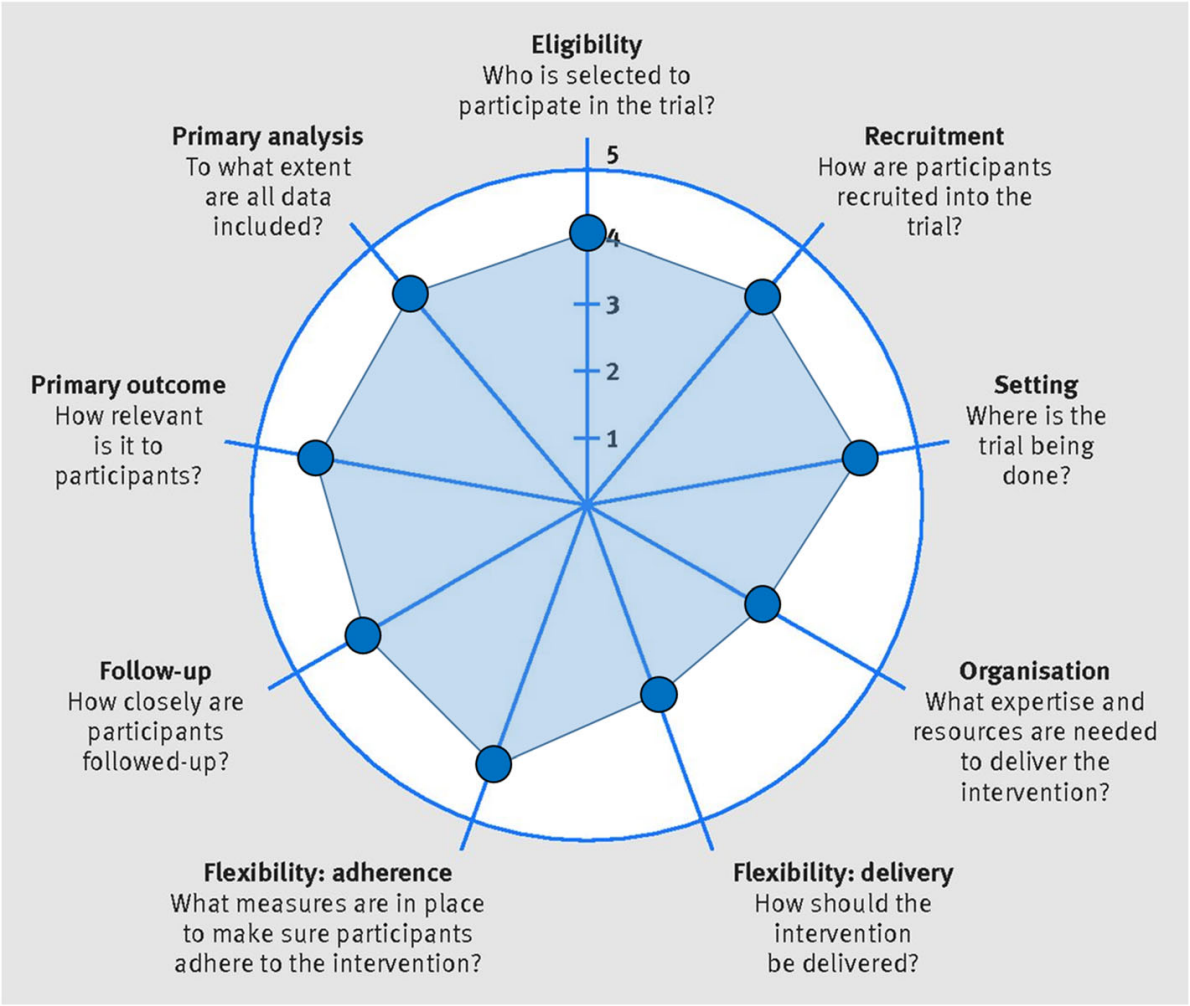
The PRagmatic-Explanatory Continuum Indicator Summary 2 (PRECIS-2) wheel [17]

**Fig. 2:**
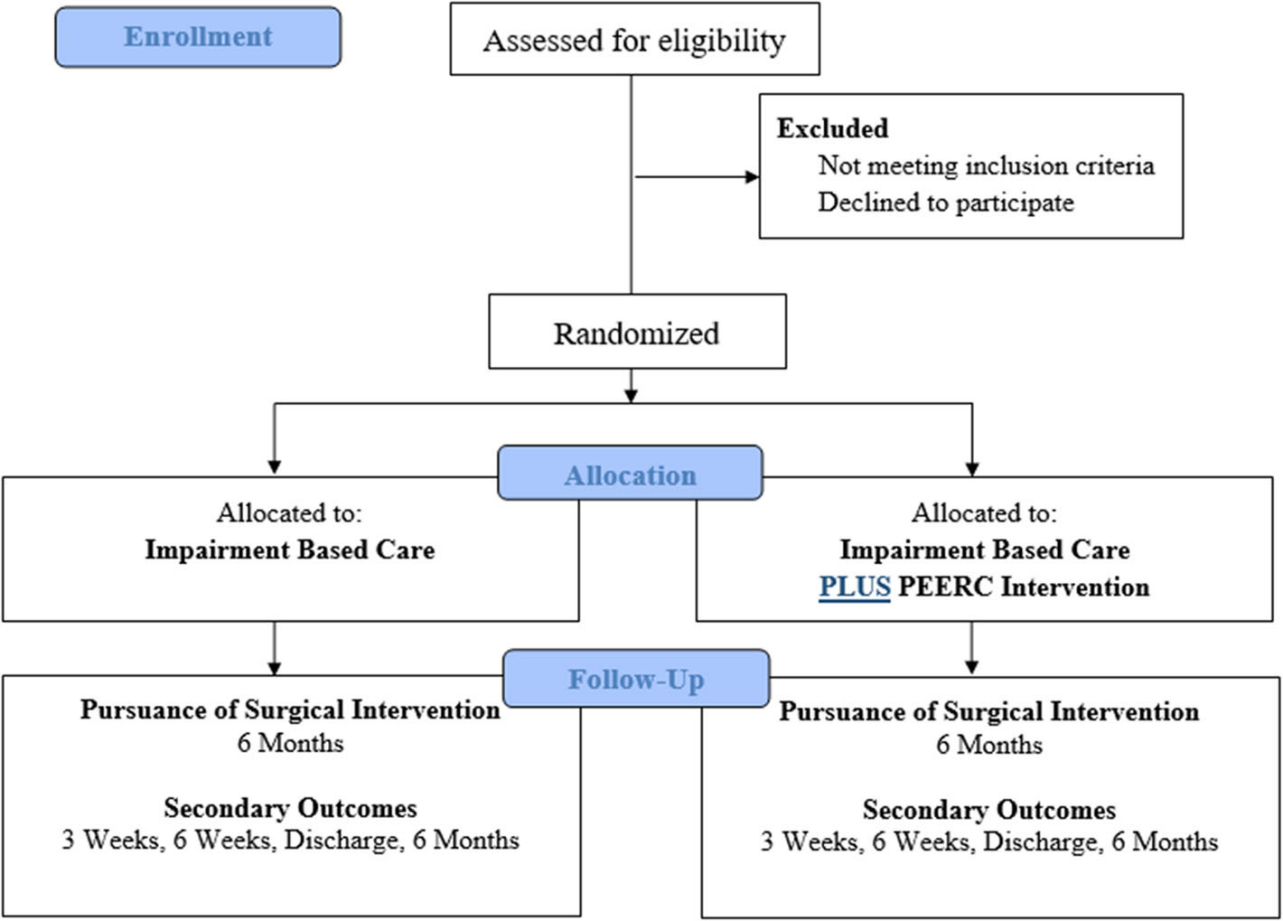
CONSORT Flow diagram for enrolment, allocation, and follow up

The study design is a randomized, pragmatic, “add-on” clinical trial. Pragmatic trials optimize normal everyday care processes and are designed to show the ‘real-world’ effectiveness of an intervention in broad patient groups. The PRagmatic-Explanatory Continuum Indicator Summary 2 (PRECIS-2) is a tool to gauge if the study design matches the intended purpose by rating nine domains on a continuum from very explanatory (ideal conditions) to very pragmatic (usual care conditions) (ICC > 0.67) [17, 18]. Fig. 1 illustrates the continuum for the PEERC trial based on input from the study team. Add-on trials are appropriate when an experimental intervention is tested on participants with a condition in which an established, effective treatment is present. In “add-on” trials, all participants (in both conditions) receive an established, effective treatment. Add-on designs are especially useful for testing of experimental interventions with mechanisms of action different from that of the established, effective treatment [19]. Add-on trials allow the investigators to better understand the isolated ‘effect’ of the “add-on” intervention [20]. In this study, the PEERC intervention will be the “add-on” component to the established impairment-based physical therapy treatment. Planning for this study was initiated in 2017 and continues with enrolment. This study protocol is described using both the SPIRIT [21, 22] guideline for the minimum content of a clinical trial protocol and the CONSORT Statement and Checklist [21] for reporting in clinical trials to facilitate complete reporting. The TIDieR [22] guidelines were followed when describing the study interventions. Figure 2 illustrates the flow of enrolment, allocation, and follow up. The trial is registered on ClinicalTrials.gov: NCT03353272.

## Methods

### Recruitment and Consent

Consecutive patients with RCRSP referred by primary care physicians, orthopaedic surgeons, and physician assistants for physical therapy are recruited for the trial. RCRSP diagnoses include conditions such as subacromial pain (impingement) syndrome, rotator cuff tendinopathy, and symptomatic partial and full thickness rotator cuff tears. The informed consent process is performed by institutionally-trained research personnel. Study activities are not initiated until after the patient provides written consent.

### Setting

All patients are treated at Duke Sports Physical Therapy at the James R. Urbaniak, MD Sports Sciences Institute at Duke University located in Durham, North Carolina, United States. This clinic employs over 30 physical therapists, the majority having advanced specialization in musculoskeletal-based disorders and experience participating in clinical trials. Several physicians, both primary care physicians and orthopaedic surgeons, from the James R. Urbaniak, MD, Sports Sciences Institute, who specialize in management of shoulder conditions, refer patients directly to this hospital-based outpatient physical therapy clinic.

### Inclusion and Exclusion Criteria

Inclusion criteria for this protocol include: ages 18 to 70; a mobile or land-line telephone; the ability to read, write, and speak English; and an RCRSP diagnosis inclusive of both acute and chronic cases for which the date of onset will be recorded. We exclude patients who have received, or are scheduled for, a surgical intervention for their shoulder condition, demonstrate any evidence of cervicogenic pain and/or radiculopathy from cervical origin, or who demonstrate symptoms consistent with thoracic outlet syndrome; all of which will be identified during the clinical examinations by the attending physician and physical therapist. We also exclude individuals who are undergoing treatment for a serious psychological disorder (e.g., severe depression, psychosis).

### Randomization and Blinding

Consented participants are randomized to receive either the (1) impairment based care group or (2) impairment based care plus PEERC group (Fig. 2). Consecutively numbered, sealed, opaque envelopes containing group allocation were prepared by a researcher with no other involvement in the study. Condition allocation involves randomization within random permuted blocks using the random number function in Excel and is stratified according to treating therapist so that all physical therapists will deliver approximately equal numbers of patients in both conditions to control for therapist variation. Participants are blinded to study purpose of improving expectations of conservative care and decisions to pursue surgery. Rather than employ deceit, it is communicated that the investigators wish to improve the patient experience through additional education and interaction.

### Interventions

Both groups receive a dedicated impairment-based, physical therapy approach that is performed in the same clinic.

Impairment-based care- The impairment based care is pragmatic, but involves an established, three step phased approach supported by Kuhn [23], Garrison [24], and Stevenson [25]. The three phases include an inflammatory phase, a subacute/early strengthening phase, and an advanced strengthening phase. Patients move from one phase to another based on report of pain and mastery of the activities within the current phase. The home exercise program of the phased approach will be standardized but the dosage of the clinical interventions will be specific to the examination findings. The phased approach allows patient-centred care that is unique to the needs of the patient and his/her progress, but reduces the variability of care that is common in physical therapy settings. Table 1 outlines the staging criteria, goals, and sample exercises of the three phases used in this protocol. Subsequent visit frequency and duration is determined pragmatically by the evaluating physical therapist and may be adjusted in response to progress toward goals. This evaluating therapist is considered the primary therapist, with a secondary physical therapist providing care in the event that the primary therapist is unavailable. No participant is treated by more than two different therapists over the course of his or her care. Participants who have received a corticosteroid injection for rotator cuff related shoulder pain will not be excluded, nor will subsequent concomitant use of injection be cause for withdrawal from the study. Corticosteroid injections, along with the date of the injection, will be recorded in the participant’s study record and in the study database. Oral NSAIDS will also be permitted at the patient’s discretion. In the absence of extenuating circumstances, patients are discharged from care when all goals (see Table 1) of each treatment phase are met.

**Table 1:** Phased Criteria for Progression of Therapeutic Exercise

Phase I: Weeks 0–2 No activities that cause pain > 3/10.	Goal: Decrease inflammation and pain Goal: Improve glenohumeral and scapulothoracic ROM Goal: Address soft tissue restriction as it relates to postural positioning (i.e. Pectoralis Minor and Posterior Capsule) Goal: Improve scapular neuromuscular control	Sample activities may include: PROM and/or AAROM activities to address range of motion deficits; Pectoralis Minor stretch on foam roll, cross body adduction stretch, thoracic mobilization on foam roll, postural corrective exercises, scapular setting with ROM activities in supine and progressing to standing; resisted scapular retraction; resisted scapular depression; resisted serratus anterior activities Criteria to progress to Phase II: - Demonstration of proper postural alignment with scapular setting - minimal upper trap activation, no scapular winging, or, if winging is present it must be asymptomatic with negative retraction and assistance tests - Able to achieve full pain free passive flexion in supine. - 80 % active flexion against gravity - worst pain 5/10 or less during normal ADLs
**Phase II: Weeks 3–5** Progress from AAROM to AROM in progressively gravity dependent positions. Closed chain exercises, resistance exercises below 90 degrees elevation, and stabilization exercises.	**Goal**: full pain free AROM in all planes with good mechanics **Goal**: Increase strength/motor control of scapular and rotator cuff muscles for active use of arm	**Sample activities may include**: 90/90 Pectoralis Minor stretch, sleeper stretch, AROM activities with focus on proper movement pattern; prone mid trap and lower trap strengthening; resisted periscapular strengthening; resisted rotator cuff strengthening below shoulder height; closed chain stabilization activities with progressive weight bearing as long as scapula is stable and not winging under load. **Criteria to progress to Phase III**: - full pain free AROM shoulder flexion with symmetric mechanics and no or asymptomatic scapular dyskinesia - 5/5 MMT of scapular and rotator cuff musculature or within 10 % of uninvolved upper extremity as measured with HHD
**Phase III**: **Weeks 6–8** Education to perform functional exercises maintaining postural awareness and scapular stabilization. Advance rotator cuff and scapular strengthening exercises.	**Goal**: Normalize rotator cuff and scapular strengthening; restore pattern-generated movements **Goal**: maintenance of posture and alignment to become subconscious **Goal**: Integrate kinetic chain activities pertinent to sport/work demands **Goal**: Return to normal function with ADLs and recreational activities	**Sample activities may include**: strengthening and stabilization activities above 90 degrees; diagonal patterns; dynamic activities to improve performance during functional and/or athletic tasks; core, balance, and endurance activities needed for work/sport **Criteria for discharge**: - Maintain full and pain-free AROM in all planes in seated or standing position with good mechanics (no or asymptomatic scapular dyskinesia) - Demonstration of 5/5 MMT or 10 % margin as measured with HHD for shoulder flexion, abduction, rotator cuff, scapular stabilizing muscles - MCIDDIC of outcome measures will demonstrate significant change in function - Patient demonstrates independence with home exercise program and strategies for self-management of symptom resolution should they arise

*ROM* Range of Motion, *AROM* Active Range of Motion, *PROM* Passive Range of Motion, *ADL *Activity of Daily Living, *MMT* Manual Muscle Test, *HHD *Hand Held Dynamometry, *MCID* Minimally Clinical Important Difference

PEERC Intervention- All patients in this condition receive the care outlined above for the impairment-based care condition. In addition, they receive a telephone-based intervention (PEERC), designed by the authors, to challenge and change underlying thoughts, beliefs, and attitudes related to treatment expectations regarding conservative care. PEERC, based on cognitive-behavioural principles, is delivered by one of two specifically trained physical therapists who conduct six 30-minute telephone sessions with participants over a six-week period beginning the second week of physical therapy participation. Treatment techniques used in PEERC are drawn from CBT to address issues related to thought distortions and irrational beliefs common in patients who have RCRSP. These techniques, summarized in Table 2, are grouped into three domains: engagement, education and cognitive structuring.

**Table 2:** Components and Defining Elements of the PEERC Intervention

PEERC Component	Defining Elements of the PEERC Intervention
**Patient Engagement**	The engagement approach uses motivational interviewing (a method that works on facilitating and engaging intrinsic motivation within the client in order to change behavior) and elicitation of thoughts and beliefs about the patient’s targeted expectations. The physical therapist uses behavioral interviewing methods to elicit information on thoughts, feelings, and behavior related to pain. The interaction focuses on the patients’ pain, how pain influences their lives, and their thoughts and beliefs about pain.
**Education**	The health coach emphasizes that pain is a complex, multidimensional experience that is affected by thoughts, feelings and behaviors as well as the important role that self-management plays in treatment. Educational methods are informed by the literature on health literacy and use simple diagrams and charts from a provided booklet to help patients understand the equivocal results of conservative and non-conservative interventions. The health coach also familiarizes patients with the current most recommended strategies for care, including any potential harms, a timeline of expectancy and recurrence rates.
**Cognitive Restructuring**	Cognitive restructuring involves learning how: (1) to recognize thoughts that are distorted, unrealistic and/or self-defeating; (2) to replace these thoughts with more rational, realistic, adaptive thinking [26]; and (3) to engage in behavioral activation assignments designed to reinforce more adaptive thinking. Home based strategies such as behaviorally testing negative thoughts via behavioral activation exercises (e.g. engaging in an activity the patient is capable of, but has been avoiding because of fear of pain) are assigned. A number of CBT methods are employed, including discussion of how thoughts, feelings, and actions affect and are affected by pain, and a review of prior session content and practices. The patient is encouraged to identify activities that the user tends to avoid, leading to discussions of how to change activities and the development of an individual plan to increase the fit between daily activities and personal goals.

### Outcome Collection

Outcomes of interest are collected by study personnel at the time of consent, after 3 weeks of intervention, after 6 weeks of intervention, at discharge, and at 6 months following discharge. With the exception of the final time point, these measures are collected in person. Table 3 illustrates the schedule of enrolment, interventions, and assessments.

**Table 3:**
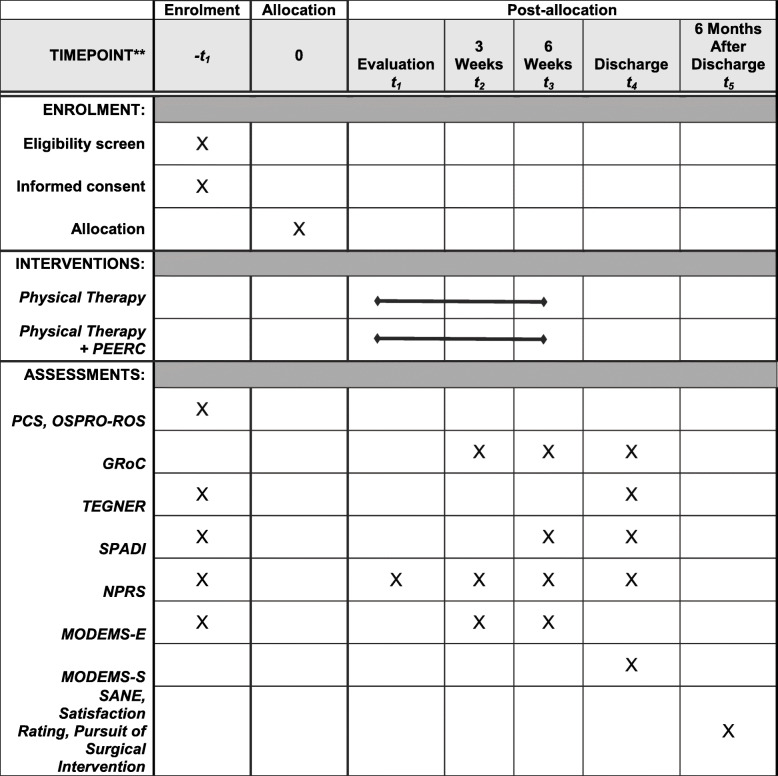
Schedule of enrolment, interventions, and assessments

### Primary Outcome

Our primary outcome measure is patient report of the decision to pursue surgery. Since the “add on” intervention of our study included patient decision-making support options, education, and facts that were designed to influence expectations, we were interested in whether this approach truly influenced high-stakes patient decisions, such as pursuance of surgery. Prior surgical decision-making studies have used similar or Likert-based outcomes [27, 28]. We selected a question framed around the patient’s choice among treatment approaches, and included the binary question: “Have you had surgery or are you scheduled for surgery for the shoulder problem that you were treated for in physical therapy?” We will use telephone contact at 6 months after discharge from impairment-based care. Because patients may pursue surgery outside of the study institution, in which case a scheduled procedure would not be documented in the medical record, this direct contact will better serve to capture decision of surgical pursuance.

### Secondary Outcomes

Secondary outcome measures include changes pain, function, expectations and satisfaction with conservative care. The construct of pain is assessed through the Shoulder Pain and Disability Index (SPADI) [29–32] and the Numeric Pain Rating Scale (NPRS) [33–35]. Functional constructs are measured with the SPADI, Tegner Activity Scale (TEGNER) [36], Single Assessment Numeric Evaluation (SANE) [37–39], and the Global Rating of Change (GRoC) [40–45] score. Expectations and satisfaction are measured with the MODEMS-E and MODEMS-S questionnaires respectively. The MODEMS [26, 46, 47] is a set of musculoskeletal assessment instruments created by the American Academy of Orthopaedic Surgeons.

Expectations - The MODEMS expectations scale (MODEMS-E) is a six-item instrument designed to capture patient expectations across a wide range of musculoskeletal conditions. The MODEMS-E patient expectation scale has been used by a number of studies and has shown validity in predicting outcomes in conservative and surgical interventions. The instrument is a Likert-based scoring tool with a mean score of 5 out of 5 (indicating high expectations of positive outcomes) and a mean score of 1 out of 5 (indicating very poor expectations of positive outcomes) [46].

Satisfaction - Patient satisfaction with the conservative care received in our clinic will be measured using the MODEMS-S. The MODEMS –S consists of five similar stated questions from the MODEMS-E, but the questions are written to assess whether one’s expectations were met. The MODEMS-S instrument is also a Likert-based scoring tool with a mean score of 1 out of 5 (indicating expectations were met) and a mean score of 5 out of 5 (indicating expectations were not met) [46]. Table 4 details further description and the psychometric properties of each patient reported outcome included in the study.

**Table 4:** Patient Reported Outcomes Measures Administered in the Study Protocol

Measure	Abbreviation	Collection Time point	Psychometrics	Construct(s)	Comments
Optimal Screening for Prediction of Referral and Outcome [48]	OSPRO-ROS	Baseline	Concurent Validity (pain) *r* = 0.31 [49]	Comorbidities, Systemic Pathology	A review-of-systems screening tool that includes constructs associated with comorbidities
Pain Catastrophizing Scale	PCS	Baseline	ICC 0.92 (95 % CI) [50, 51]	Catastrophizing (rumination, magnification, helplessness)	13-item questionnaire that assesses the degree to which individuals have different thoughts and feelings when in pain.
Tegner Activity Scale [52]	TEGNER	Baseline and Discharge	ICC = 0.80 MDC- 1.0 [36]	Function	Function and activity as a numerical value between 0 (complete disability) to 10 (elite athletics)
Shoulder Pain and Disability Index [29]	SPADI	Baseline, 6 weeks, Discharge	ICC – 0.96 [30]CV (Global Disability Rating) – 0.64[32]MDC- 21.5 [30]MCID- 15.4 [31]	Pain and Function	Assess two domains; a 5-item subscale that measures pain and an 8-item subscale that measures disability
Numeric Pain Rating Scale	NPRS	Baseline, 3 weeks, 6 weeks, Discharge	IC – 0.88 [34]Excellent Intra-rater and Interrater reliability with 100 % agreement [34]CV (VAS)- r = 0.94; 95 % CI = 0.93–0.95 [35]MCID- 2.17 [33]	Pain	0 to 10 (11 point scale) based on pain intensity with 0/10 representing no pain and 10/10 representing the worst pain possible
Musculoskeletal Outcome Data Evaluation Management System [46]^,^[47]ExpectationsSurvey &Satisfaction Survey*	MODEMS-E MODEMS-S	Expectations: Enrollment, 3 weeks, 6 weeksSatisfaction:Discharge	Reliability Cohen’s kappa 0.91 [26]Internal consistency Cronbach’s alpha 0.71 [26]	Patient Expectations	Likert-based scoring tool with calculated mean score
Global Rate of Change [53]	GROC	3 weeks, 6 weeks, Discharge	ICC 0.9 [41]; face validity between GRC and patient ratings of the importance of change (Pearson’s r = 0.90) [42] and patient satisfaction measures (Spearman’s rho 0.56–0.70) [43] CR - Shoulder Disability Questionnaire r = 0.74) [44], NPRS (r = 0.49) [45]	Therapeutic Outcome	Global rating of change relative to baseline using a 15-point ordinal scale (where − 7 is much worse and + 7 is much better)
Single Assessment Numeric Evaluation [54]	SANE	6 months post discharge	ICC 0.84 [39]Correlation with ASES (Spearman’s Rho 0.77) [38]MCID 15 % [39]	Function	“Please rate your shoulder function on a scale of 0 % (no function) to 100 % (full, normal function)."

### Patient Demographics and Characteristics

To describe patient demographics and presentation, we will report age, sex, education level, work status, marital status, referral source (primary care physician or orthopaedist), history of corticosteroid injection for the current episode, and prior participation in physical therapy for same or different diagnosis. Instruments to assess systemic and comorbidities include the Optimal Screening for Prediction of Referral and Outcome- Review of Systems (OSPRO-ROS) [48] and the Pain Catastrophizing Scale (PCS) [50, 51]. The OSPRO-ROS is a review-of-systems screening tool that includes constructs associated with comorbidities and systemic pathologies [55]. The PCS is a 12 item questionnaire ranking types of thoughts and feelings one has while in pain from 0 (not at all) to 4 (all the time) [50]. Table 4 details the psychometric properties of the OSPRO-ROS and PCS.

### Sample Size Estimate

We powered the study for proportional differences between conditions on decision to have surgery for up to 6 months. Using projections from previous data, and assuming offset inequity between two independent conditions; we modelled power on the following assumptions. In the absence of prior studies, the authors project 30 % of the impairment-based condition only to pursue surgery versus 5 % from the impairment-based plus PEERC condition. This assumes an allocation ratio of 1/1 and error of probability of 0.05 and projected power of 80 %. With these assumptions, our projected sample size requires 94 participants. We will employ intention to treat in the primary analysis and do not plan for dropouts.

### Statistical Analysis

We will evaluate descriptive statistics of the two conditions using appropriate parametric and nonparametric tests for differences, depending on the data (continuous or frequency based). For our primary outcome (patient-reported surgery or intention to have surgery), we will measure condition differences in proportions between the impairment-based care only shoulder treatment and the impairment-based care plus PEERC, using a chi-square analysis (or Fisher Exact).

For our secondary aims, we will use linear mixed effects modelling to compare pain (NPRS and SPADI), function (SPADI, GRoC, TEGNER, and SANE), and follow up expectations and satisfaction (MODEMS) between the two conditions. Linear mixed effects modelling methods are flexible, model individual change, and accommodate for missing data (when present). We will run two analyses, unadjusted and adjusted, in which we will control for all baseline characteristics that are significantly different (if present) and baseline patient expectation, functional outcome, and pain. Pain intensity measures will be evaluated using a negative binomial Poisson, which accounts for count variables with significant skew.

### Data Collection and Management

Study data are managed using REDCap (Research Electronic Data Capture) [56] electronic data capture tools hosted by the study institution. REDCap is a secure, web-based platform designed to provide an interface for validated data capture and export of data to statistical packages. De-identified data, both from the secure electronic medical record as well as paper questionnaires, is entered into the REDCap instrument by study personnel. Ownership of the final dataset rests with the institution.

### Monitoring

Because this investigation presents less than minimal risk or psychosocial harm, an independent data monitoring committee is not required. Adherence to the impairment based treatment intervention will be monitored via checklist for treatment fidelity by non-treating study personnel. To enhance treatment fidelity, the study therapists underwent a formal training program, use a treatment manual to guide their sessions. Participant retention is promoted through contact between the physical therapist and the patient along with participant honorarium provided at the initial physical therapy evaluation and at the conclusion of ten weeks of active participation.

### Ethics and Financial Support

This trial has the approval of the Institutional Review Board of Duke University under protocol identification number Pro00088103. Unanticipated problems involving risks to participants or others; information that indicates an adverse change to the risks or potential benefits of the research; or a protocol departure that harmed participants or others or compromises the integrity of the research data require prompt reporting to the institutional review board. This study is externally funded by the Academy of Orthopaedic Physical Therapy. The role of this funding source is solely financial and not influential or contributory to design or interpretation of results. All results from the study will be submitted for publication in peer-reviewed scientific journals. Prior to publication, the authors expect to present the results at professional conferences. For all forms of publication, the authors must meet the four tenets of authorship set forth by the International Committee of Medical Journal Editors. The authors declare no competing interests.

## Discussion

This trial aims to measure effects of a novel PEERC intervention designed to change expectations of conservative care. In the primary analysis, the efficacy of PEERC will be assessed via participant report of their having had or have the intent to have surgery 6 months after finishing impairment-based care. In secondary analyses we will also investigate the effect of PEERC on pain, function, expectations and satisfaction with conservative care. Reliance on patient reported outcome measures, because they often lack evidence of validity, is a recognized limitation of clinical trials. Our primary outcome, pursuance of surgery, is no exception. The medical record can be queried, but this would only serve to verify the presence, not the absence, of planned or performed surgery because the patient may seek care outside of the study institution.

When considering the management decisions for RCRSP, clinicians, policy makers, and patients are faced with an intriguing dilemma. Despite higher risks/harms with surgical treatments, conservative treatments have similar outcomes [1–4]. The comparable results may be a reflection of a dedicated focus toward a biomedically oriented strategy [57]. Patient expectations (outside the biomedically oriented mechanisms associated with conservative and non-conservative treatment approaches) may also influence functional outcomes and the decision to pursue additional care options. This assumption is supported by studies that have identified the prognostic role of patient expectations across a variety of conservative and non-conservative care options [47, 58].

A patient-centered approach emphasizes patients’ treatment expectations - i.e. their desires, thoughts, and beliefs about treatment and its outcome [59]. Patient-centered models of care allow patients more control in directing their treatment and are a viable alternative method of enhancing treatment outcomes [60]. No studies of RCRSP have investigated the influence of brief, theory-based interventions for changing expectations about what treatment is appropriate/optimal when evidence suggests there is not an obvious choice based on superiority. Our study will focus on addressing patient expectations to understand that a conservative approach will provide a similar outcome to surgery, without unnecessary risks.

Despite recognition that patient expectations has a role in influencing functional outcomes and the decision to pursue surgical care in patients with RCRSP [8–10], there have been few studies designed to test interventions designed to change expectations [15, 16]. Research has shown that the best predictor of functional outcomes with a conservative approach are pre-treatment, patient expectations [15, 61]. Expectations (i.e. attitudes, thoughts, and beliefs) are malleable using methods rooted in cognitive-behavioral science that focus on education, engagement and cognitive restructuring.

If this innovative PEERC intervention is successful, the approach may provide the appropriate foundations that could be applied to other musculoskeletal disorders where there are viable conservative options to surgical care.

### Trial Status

Recruitment of participants began on September 18, 2018 and currently continues. Completion is anticipated at the conclusion of 2021. The final decision to terminate the trial rests with the primary investigator.

## Data Availability

Not applicable.

## Abbreviations

PEERC: Patient Engagement Education and Restructuring of Cognitions
CBT: Cognitive Behavioral Therapy
RCRSP: Rotator Cuff Related Shoulder Pain
MODEMS: Musculoskeletal Outcome Data Evaluation Management System
SPADI: Shoulder Pain and Dysfunction Index
OSPRO-ROS: Optimal Screening Prediction of Referral and Outcome
PCS: Pain Catastrophizing Scale
SANE: Single Assessment Numeric Evaluation
TEGNER: Tegner Activity Scale
NPRS: Numeric Pain Rating Scale
VAS: Visual Analog Scale
GRoC: Global Rate of Change
